# Ultrathin MoS_2_ Nanosheets with Superior Extreme Pressure Property as Boundary Lubricants

**DOI:** 10.1038/srep12869

**Published:** 2015-08-07

**Authors:** Zhe Chen, Xiangwen Liu, Yuhong Liu, Selda Gunsel, Jianbin Luo

**Affiliations:** 1 State Key Laboratory of Tribology, Tsinghua University, Beijing 100084, P. R. China; 2 Department of Chemistry, Tsinghua University, Beijing 100084, P. R. China; 3 Shell Global Solutions (US) Inc, Westhollow Technology Center, Houston TX 77210, USA

## Abstract

In this paper, a new kind of oil-soluble ultrathin MoS_2_ nanosheets is prepared through a one-pot process. A superior extreme pressure property, which has not been attained with other nano-additives, is discovered when the nanosheets are used as lubricant additives. The as-synthesized MoS_2_ nanosheet is only a few atomic layers thick and tens of nanometers wide, and it is surface-modified with oleylamine so it can be well dispersed in oil or lubricant without adscititious dispersants or surfactants. By adding 1 wt% ultrathin MoS_2_ nanosheets, at the temperature of 120 °C, the highest load liquid paraffin can bear is tremendously improved from less than 50 N to more than 2000 N. Based on the tribological tests and analysis of the wear scar, a lubrication mechanism is proposed. It is believed that the good dispersion and the ultrathin shape of the nanosheets ensure that they can enter the contact area of the opposite sliding surfaces and act like a protective film to prevent direct contact and seizure between them. This work enriches the investigation of ultrathin MoS_2_ and has potential application in the mechanical industry.

Ultrathin MoS_2_ has attracted great interest in the past few years due to its application in the fields of catalysis for hydrogen evolution reaction, new generation of semiconductors and high rate lithium battery^1^,^2^,^3^. Moreover, MoS_2_ is an excellent solid lubricant due to the easy interlayer sliding^4^,^5^. Recently, with the development of nanotechnology, MoS_2_ nanoparticles with various sizes and morphologies were prepared and they are proved to be good lubricant additives^6^,^7^,^8^,^9^. As is acknowledged, the lubrication property of MoS_2_ largely depends on its structure^10^,^11^. Although many kinds of multilayer MoS_2_ nanoparticles have been investigated, there have been only a few studies of the tribological property of ultrathin MoS_2_ nanosheets with single or few layers. However, it is a challenge to prepare appropriate ultrathin MoS_2_ nanosheets for use as lubricant additives, because ultrathin MoS_2_ nanosheets synthesized with existing methods have relatively large size and poor dispersion in lubricants^12^,^13^,^14^,^15^. Aiming at this problem, here we report a one-pot wet chemistry route to prepare a new kind of oil-soluble ultrathin MoS_2_ nanosheets. The as-synthesized sample is analysed with X-ray photoelectron spectroscopy (XPS), transmission electron microscopy (TEM), Raman spectroscopy, fourier transform infrared spectroscopy (FTIR) and the technique of dynamic light scattering (DLS). Then the extreme pressure property of the lubricant containing the as-synthesized ultrathin MoS_2_ nanosheets is evaluated with a load-climbing tribological test. Through the comparision with lubricants containing other nano-additives or organic additives, it is found that the ultrathin MoS_2_ nanosheets have superior ability of improving the extreme pressure property of the lubricant. The wear scar is analysed with scanning electron microscope (SEM), energy dispersive spectrometer (EDS) and XPS. Finally, a lubrication mechanism is proposed based on the tribological tests and surface analysis.

The ultrathin MoS_2_ nanosheets were prepared as described below (Methods). XPS results, which are shown in Fig. 1a, prove that the as-synthesized material is MoS_2_ and other compounds such as Mo_2_S_5_, MoS_3_ or MoO_3_, whose Mo3d peaks have higher binding energies, can be excluded^16^,^17^. The UV-vis spectrum of the as-synthesized MoS_2_ is presented in Supplementary Fig. S1^18^,^19^. The TEM image is displayed in Fig. 1b and there are many dark lines in the image. It looks very similar with the images obtained by Altavilla *et al.* and Grossiord *et al.*^15^,^20^ Both of them regarded the dark lines as standing parts of MoS_2_ layers. The details of the dark line (see Supplementary Fig. S2) and an observation of natural MoS_2_ through TEM (see Supplementary Fig. S3) both support this conclusion. Given that there is no parallel dark lines observed in the TEM image, one preliminary conclusion is drawn that the as-synthesized MoS_2_ is of single layer structure. In addition, it can be found through TEM that the as-synthesized MoS_2_ is in the morphology of nanosheets (see Supplementary Fig. S4). To further confirm the layer number of the as-synthesized MoS_2_ nanosheets, the sample was analysed with Raman spectroscopy. In the Raman spectrum of MoS_2_, the relative shift between the two peaks of 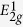 and 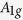 will shrink as the number of MoS_2_ layers decreases^21^,^22^. Although previous related works do not provide a uniform value of the relative peak shift for certain layered MoS_2_ and some work found that the substrate will slightly affect both the peak shift and the relative peak shift, the difference between few layer and multi layer can be distinguished^21^,^22^,^23^. For the Raman spectrum of the as-synthesized MoS_2_ nanosheets, as depicted in Fig. 1c, the relative shift between the two peaks is 19.9 ± 0.6 cm^−1^, which confirms that the as-synthesized MoS_2_ nanosheets are of ultrathin shape with only single or few layers. Moreover, it was found that the as-synthesized MoS_2_ nanosheets can be well dispersed in cyclohexane forming stable and black colored translucent solution (see Fig. 1d). Considering the fact that clean or unmodified MoS_2_ will sink in cyclohexane, it can be deduced that the MoS_2_ nanosheets have been surface-modified. And the modifier is proved to be oleylamine with the help of XPS and FTIR (see Supplementary Fig. S5)^16^,^24^. Moreover, it is believed that the amine groups attach on the MoS_2_ nanosheets through electrostatic force, because the MoS_2_ surface is negatively charged (see Supplementary Fig. S6)^25^,^26^,^27^,^28^, while the amine group is positively charged^29^. Because of the good dispersibility in cyclohexane, the particle size distribution of the as-synthesized MoS_2_ nanosheets can be obtained by the technique of DLS after adequate ultrasonic treatment and it is shown in Fig. 1e. It tells that the hydrodynamic diameters of the MoS_2_ nanosheets are in the range from about 20 nm to 150 nm and concentrated at 37.4 ± 2.0 nm. After all the analysis above, it can be concluded that the MoS_2_ nanosheets we prepared have only single or few layers with its lateral size at tens of nanometers and its surface is modified with oleylamine, as illustrated in Fig. 1f.

To investigate the lubrication property of the as-synthesized ultrathin MoS_2_ nanosheets as lubricant additives, they were dispersed in liquid paraffin (LP), which is a common base oil and whose dynamic viscosity is only 9.94 ± 0.31 cP at 50 °C and 2.28 ± 0.29 cP at 120 °C. A load-climbing test, whose details are described below (Methods), was adopted to fully evaluate the ability of the as-synthesized ultrathin MoS_2_ nanosheets to enter the contact area under high load. Together with the nanosheets, molybdenum dialkyldithiophosphate (MoDDP), inorganic fullerene-like MoS_2_ nanoparticles (IF MoS_2_) and micro-sized MoS_2_ powder (micro MoS_2_) were tested for comparision. MoDDP is a commonly used commercial lubricant additive. Under high pressure and high temperature, MoDDP will decompose forming nano-sized multilayer MoS_2_ (see Supplementary Fig. S7), but the synthesis of MoDDP is rather complex and the cost is high^30^,^31^,^32^. Since there were four different kinds of Mo-contained substance and the weight fraction of Mo in each of these substances are different, the final weight fraction of the element of Mo in the lubricant was controlled at 6 wt‰, meaning that the corresponding weight fraction of MoS_2_ was 1 wt%, which is a common concentration reported in similar researches^9^,^33^,^34^. Besides those lubricants, oleylamine was tested as well. The test temperature was set at 120 °C, which is very normal in automobile engines. The COF and load as functions of time are displayed in Fig. 2a and the highest load with no seizure of each sample is plotted in Fig. 2b.

It is quite interesting that, the tribological test with the lubricant containing the as-synthesized ultrathin MoS_2_ nanosheets did not stop until the load reached 2000 N, which is the highest load the tribotester can provide, which means that the highest load with no seizure of the lubricant is no less than 2000 N. The COF of the lubricant containing MoDDP rose abruptly when the load reached 700 N, indicating that the highest load with no seizure is 600 N. The highest load of the lubricant containing IF MoS_2_ is only 300 N. The performance of the lubricant containing micro MoS_2_ is the worst and 200 N is its limit. The lubricant containing the as-synthesized ultrathin MoS_2_ nanosheets distinguishes itself from other tested lubricants that its highest load with no seizure is at least three times bigger than that of others. Besides, the highest load with no seizure of oleylamine is only 300 N, whichs rule out any possibility that oleylamine is the reason of the superior lubrication property. Furthermore, tribological tests with various Mo weight fraction at different temperatures (see Supplementary Fig. S8) showes that the as-synthesized MoS_2_ nanosheets performed better than MoDDP under all the tested conditions. In addition, graphene, triphenyl phosphorothionate (TPPT) and zinc dithiophosphate (ZDDP), the later two of which are commonly used organic lubricant additives and are introduced in Supplementary Fig. S9, were evaluated under the same conditions. None of the lubricants containing these additives can bear more than 400 N (see Supplementary Fig. S10).

After the tribological test lubricated with the lubricant containing the as-synthesized MoS_2_ nanosheets stopped at 2000 N, the wear scar was ultrasonicly cleaned and analysed. The SEM image is provided in Fig. 3a. The width of the wear scar is about 1.39 ± 0.06 mm, indicating that the final pressure is about 1.32 GPa, which is 37.5% bigger than that of MoDDP (see Supplementary Fig. S11). EDS analysis (see Fig. 3b) shows that the elements of Mo and S can be detected only inside the wear scar and XPS analysis (see Fig. 3c) further proves that the elements of Mo and S are in the chemical state of MoS_2_^16^. Therefore, it can be concluded that a tribofilm made of MoS_2_ was formed on the wear scar.

From the absolute value of COF, it can be deduced that the contribution of interlayer sliding for the lubrication is limited. It is consistent with the work of Lee *et al.* and Deng *et al.* that the friction force will increase as the layer number decreases^10^,^35^. And it is due to the local distortion of the single- or few-layered nanosheets. Although in this condition, the ultrathin MoS_2_ nanosheets will increase the interlocking friction between the sliding surfaces as the ‘third bodies’^36^,^37^, it is believed that they act more like a protective film to seperate the opposite peaks from direct contact. As is reported, the Young’s modulus and breaking strength of monolayer MoS_2_ is much larger than those of steel^38^. In addition, the simulation from Klemenz *et al.* provides further evidence that 2D-material can be an excellent coating for low friction and wear as long as the 2D-material itself is not damaged^39^. According to the analysis, the ability for 2D-material nano-additives to enter and stay in the contact area is the core problem. Fortunately, the as-synthesized MoS_2_ nanosheets have this ability due to its unique characters. Firstly, the befitting surface modification makes them floated seperately and freely in the lubricant without agglomeration. Secondly, the extreme tiny thicknness makes them easy to enter the contact area. As shown in Fig. 4a, when one micro peak is moving to the other one, the ultrathin MoS_2_ nanosheets will enter the contact area instead of being pushed away. When the two opposite peaks touch each other, because of the ultrathin MoS_2_ nanosheet between them, they will not contact with each other directly. They may be deformed due to pressure but little wear will be made because of the protection from the ultrathin MoS_2_ nanosheet. After the two peaks leave each other, the nanosheet may adhere to one of the peaks or floate back into the lubricant. This process is taking place countless times at the same moment during the test, because countless micro peaks from different sliding surfaces are meeting and seperating. As long as most of the contact peaks do not contact directly, the lubrication will not fail and the operation of the test will not stop. As for the ordinary 3D nanoparticles, such as the IF MoS_2_ or other multi-layer MoS_2_ nanoparticles, because of the relatively big size, they are more likely to be pushed away and opposite peaks will contact directly resulting in wear or seizure under high load (see Fig. 4b).

In summary, we have prepared a novel kind of oil-soluble ultrathin MoS_2_ nanosheets, which have only single or few atomic layers with its lateral size of tens of nanometers. Befitting surface modification was made through the synthesis process, so the ultrathin MoS_2_ nanosheets can be well dispersed in oil or lubricant without any extra surfactants or dispersants. The ultrathin shape of the nanosheets, together with the good dispersion in lubricant, makes them very easy to enter the contact area and prevent direct contact of the sliding surfaces under high load. As a result, the as-synthesized MoS_2_ nanosheets, when dispersed in lubricant, can largely enhance the extreme pressure property of the lubricant and have potential application for mechanical equipment operated in harsh conditions to avoid seizure or jam.

## Methods

### Synthesis

In a typical synthesis route of the ultrathin MoS_2_ nanosheets, 0.05 mmol of ammonium molybdate and 0.1 mmol of thiourea were added into the solution with 15 ml 1-octadecene and 15 ml oleylamine. After 15 minutes with vigorous stirring, the mixture was transferred to a teflon-lined stainless autoclave. The reaction lasted for 12 hours at 230 ^°^C. After that, the autoclave was taken out to cool down slowly to the ambient temperature. The products were collected and washed with 30 ml ethanol for 3 times. Then the samples were dried in vacuum at 40 °C for 2 hours.

### Tribological Tests

All the lubrication tests were performed with a commercial tribotester (Optimal SRV4) and the mode of the test is reciprocating ball-on-disk. The disk is fixed and the ball is pressed on the disk doing reciprocating motion with the stroke of 2 mm and the frequency of 50 Hz. During the load-climbing test, the normal load is firstly kept at 50 N for 30 seconds for running-in, and then the load rises to 100 N and keeps for 15 minutes. After that, the load is increased by 100 N every 2 minutes and the test will stop when the COF increases abruptly over 0.3, which indicates that the lubrication has failed and the ball and the disk have contacted with each other directly and seizure between them has taken place. The load before the lubrication fails is referred as the highest load with no seizure of the lubricant. For each test, a new ball and an un-rubbed position of the disk were put into use. All the tribological tests were performed several times with at least three tests having the same result to ensure that the value of the highest load is correct. The material of both balls and disks are bearing steel (AISI 52100) and the diameter of the ball is 10 mm. The ball is a commercial product and the surface roughness (Ra) is about 18.5 nm. The surface of the disk was polished and the roughness (Ra) is about 20.4 nm. The surface roughness of both ball and disk was measured by a commercial surface mapping microscope (ADE PHASE SHIFT MicroXAM). The viscosity of liquid paraffin was measured three times with a standard rheometer (Anton Paar Physica MCR301).

### Characterization and Analysis

Chemical configurations were determined by an X-ray photoelectron spectroscope (Thermo Fisher ESCALAB 250Xi). XPS measurements were performed with an Al K α X-ray source on the samples. The energy calibrations were made against the C1s peak. A silicon chip was used to hold the sample. UV-vis spectrum was obtained with a commercial ultraviolet-visible spectrophotometer (Hitachi U-3010). Transmission electron microscope (JEOL JEM-2010, operated at 120 kV) was used to obtain the morphology of as-synthesized samples. Raman spectra with the resolution of 0.7 cm^−1^ were obtained after all the processes introduced in the Synthesis section. On that occasion, the as-synthesized MoS_2_ nanosheets were aggregate forming small black solid blocks. Laser was focused on the surface of these blocks. Three different locations, which were chosen randomly, were analysed. A confocal Raman microscopic systems (HORIBA Jobin Yvon HR800) was used. The spectrometer used the 514 nm line of an argon ion laser and it was calibrated with the Si peak at 520.7 cm^−1^. Particle size distribution and zeta potential was obtained with a commercial zetasizer (Malvern Nano-ZS). The infrared spectra were obtained from a commercial FTIR Spectrometer (Thermo Scientific Nicolet iS10). The wear scar on the disk was analysed with scanning electron microscope (FEI Quanta 200 FEG) equipped with an energy dispersive spectrometer (EDAX Genesis).

## Additional Information

**How to cite this article**: Chen, Z. *et al.* Ultrathin MoS_2_ Nanosheets with Superior Extreme Pressure Property as Boundary Lubricants. *Sci. Rep.* **5**, 12869; doi: 10.1038/srep12869 (2015).

## Supplementary Material

Supplementary Information

## Figures and Tables

**Figure 1:**
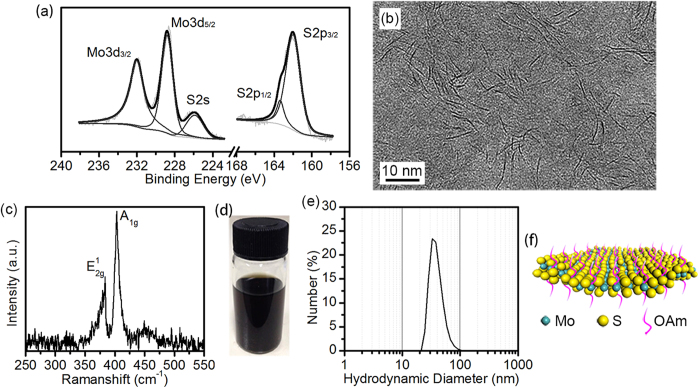
The characterizations and thus speculative structure of the as-synthesized ultrathin MoS_2_ nanosheets. (**a**) The Mo3d XPS spectrum and S2p XPS spectrum of the as-synthesized MoS_2_ nanosheets with Mo3d_3/2_ at 232.0 eV, Mo3d_5/2_ at 228.8 eV, S2s at 226.0 eV, S2p_1/2_ at 163.3 eV and S2p_3/2_ at 162.0 eV. (**b**) The TEM image of the as-synthesized MoS_2_ nanosheets and each of these dark lines is a standing part of one MoS_2_ layer. (**c**) The Raman spectrum of the as-synthesized MoS_2_ nanosheets with peak 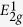 at 383.6 ± 0.4 cm^−1^ and peak 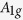 at 403.2 ± 0.5 cm^−1^. (**d**) The black colored translucent solution with the as-synthesized MoS_2_ nanosheets dispersed in cyclohexane. (**e**) The particle size distribution of the as-synthesized MoS_2_ nanosheets. (**f**) The atomic illustration of the as-synthesized MoS_2_ nanosheet.

**Figure 2:**
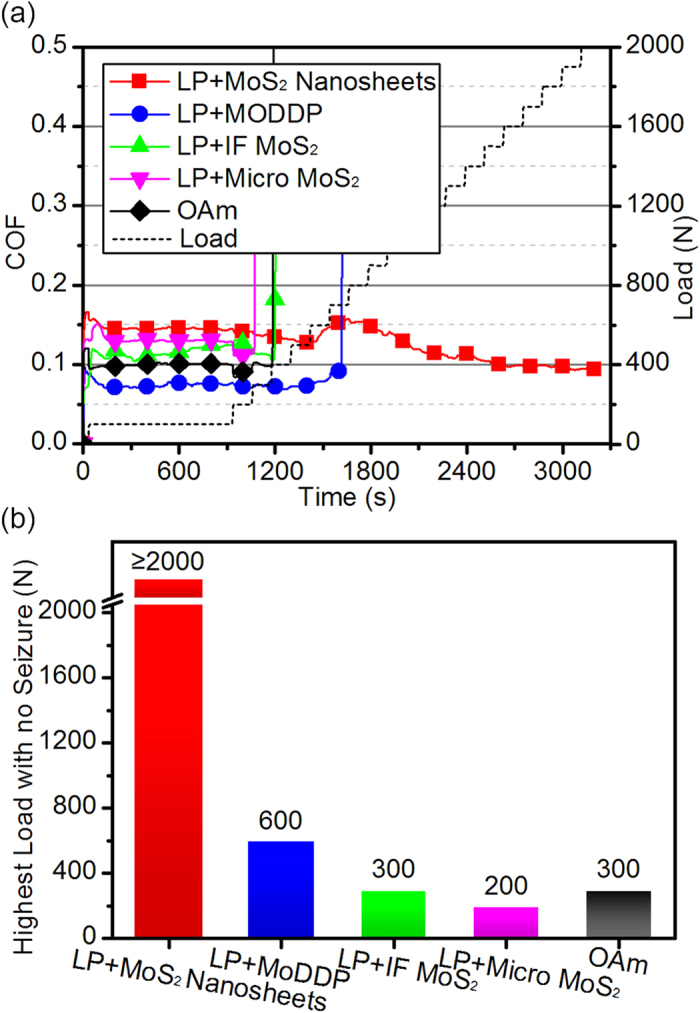
The results of the load-climbing tribological tests. The tested samples include four lubricants, which contain the as-synthesized ultrathin MoS_2_ nanosheets, MoDDP, IF MoS_2_ and micro MoS_2_ respectively, and oleylamine. The weight fraction of element of Mo in each lubricant was controlled at 6 wt‰. (**a**) COF and load as functions of time of the tests. The COF of repeated tests fluctuates within 0.01. (**b**) Highest load with no seizure of these lubricants and oleylamine (OAm).

**Figure 3:**
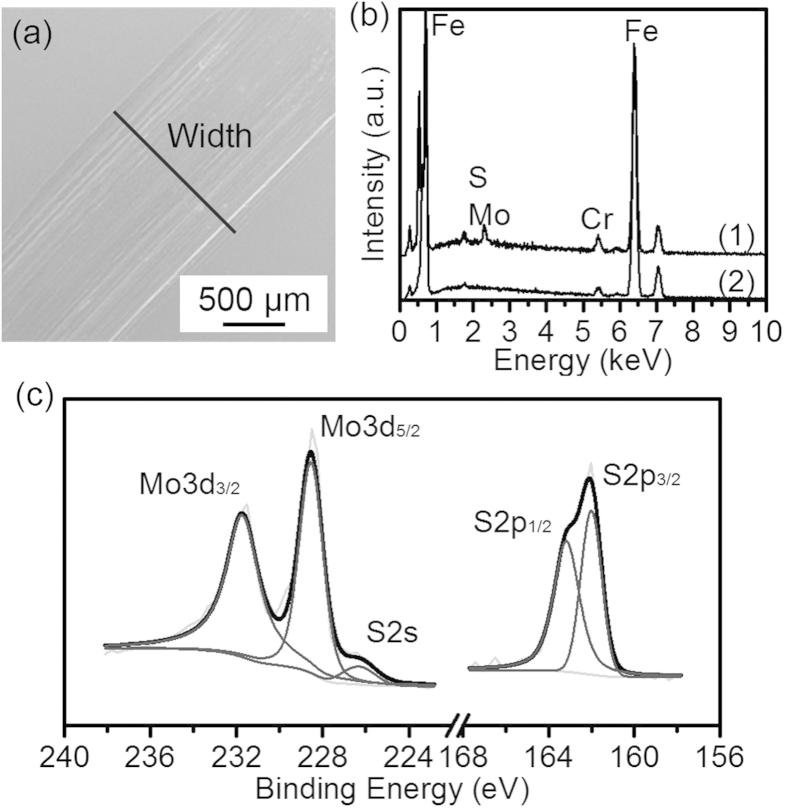
The analysis of the wear scar of the test of the lubricant containing the as-synthesized MoS_2_ nanosheets stopped at 2000 N. (**a**) The SEM image of the wear scar and the width is about 1.39 ± 0.06 mm. (**b**) The EDS spectra (1) inside and (2) outside the wear scar, and the elements of Mo and S can only be detected inside the wear scar. (**c**) XPS analysis of the elenents of Mo and S in the wear scar, with Mo3d_3/2_ at 231.7 eV, Mo3d_5/2_ at 228.6 eV, S2s at 226.3 eV, S2p_1/2_ at 163.2 eV and S2p_3/2_ at 162.0 eV.

**Figure 4:**
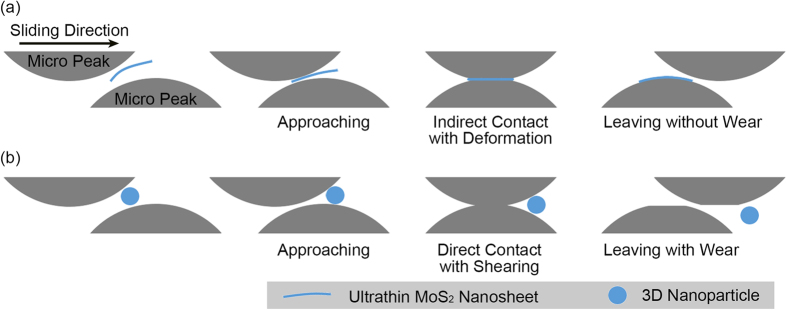
Schematics of the lubrication mechanism of (**a**) ultrathin MoS_2_ nanosheets and (**b**) 3D nanoparticles. (**a**) The nanosheet can enter the contact area of the opposite micro peaks easily and prevent them from direct contact. The peaks may deform but little wear will take place. (**b**) As for the 3D nanoparticle, it will be pushed away due to its relatively big size and opposite peaks will directly contact, resulting in wear.
