# Supplementary material for: Ultrathin MoS2 Nanosheets with Superior Extreme Pressure Property as Boundary Lubricants
Source: Sci Rep. 2015 Aug 7;5:12869. doi: 10.1038/srep12869 (PMC4528196; doi:10.1038/srep12869)
Supplement: Supplementary Information [file srep12869-s1.pdf]

Supplementary Information

Ultrathin MoS_2 Nanosheets with Superior Extreme Pressure Property as Boundary Lubricants

Zhe Chen, Xiangwen Liu, Yuhong Liu, Selda Gunsel & Jianbin Luo

1. UV-vis spectrum of the as-synthesized MoS_2.

When the as-synthesized MoS_2 was dispersed in cyclohexane, the UV-vis spectrum was obtained and is shown in Supplementary Fig. S1. It can be found that the characteristic extinctions of pristine 2H-MoS_2 in the range of 600-700 nm is not very obvious, but the high energy excitonic features in the near-UV range (200-300 nm) is quite distinct.[S1] The result is almost the same with that of Goki Eda et al.[S2] According to the analysis of Goki Eda et al., although the characteristic extinctions of pristine 2H-MoS_2 is not very clear, the MoS_2 is not totally of 1T-type and about 50% of the analyzed MoS_2 is of 2H-type. Therefore, it is believed that the as-synthesized MoS_2 nanosheets are consist of both 1T-MoS_2 and 2H-MoS_2. Moreover, XPS analysis (see Figure 1a) also supports this conclusion.[S2]

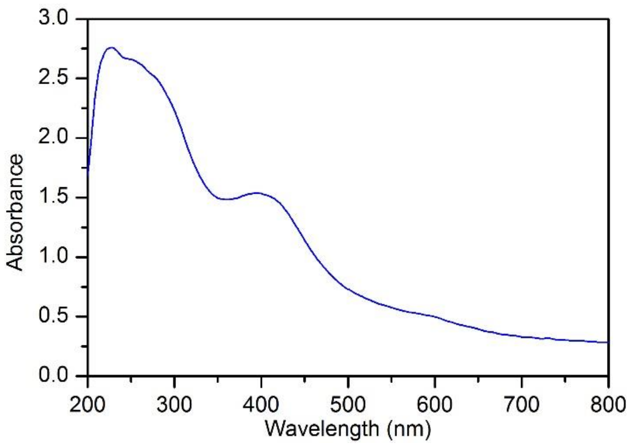

Figure S1. UV-vis spectrum of the as-synthesized MoS_2 dispersed in cyclohexane.

2. Details of the as-synthesized MoS_2.

Fig. 1b is the TEM image of the as-synthesized MoS_2 nanosheets and there are a lot of dark lines in the image. To further investigate the structure, one of the dark lines was magnified and is displayed in Supplementary Fig. S2. Clear crystal lattice can be observed in the dark line. The width of the dark line is about 0.42 nm and the lattice distance is about 0.26 nm. Given that the width of the dark line is in accordance with the thickness of one MoS_2 layer and the observed crystal lattice distance is the same with the distance between (101) planes of MoS_2, it is further proved that each of these dark lines is a standing part of one MoS_2 layer.

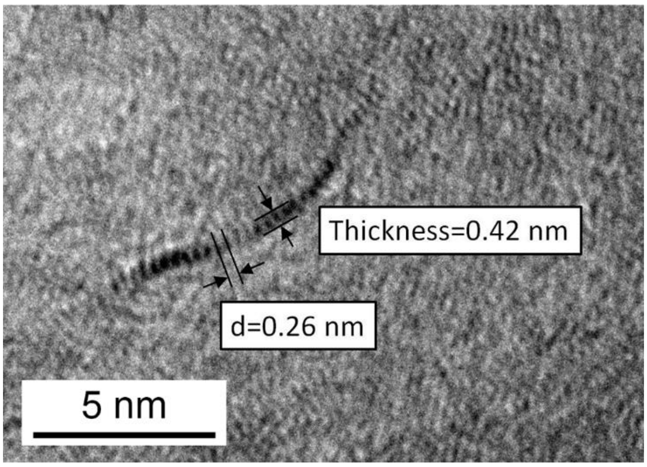

Figure S2. The details of the dark line in TEM image.

3. TEM image of natural MoS_2

Supplementary Fig. S3 shows a TEM image of a part of a micro-sized natural MoS_2 sheet. It can be seen that a part of the MoS_2 sheet's edge stands up and one dark line appears. The dark line is similar with the dark line in Supplementary Fig. S2. And it further proves that the dark line is the standing part of the MoS_2 layer.

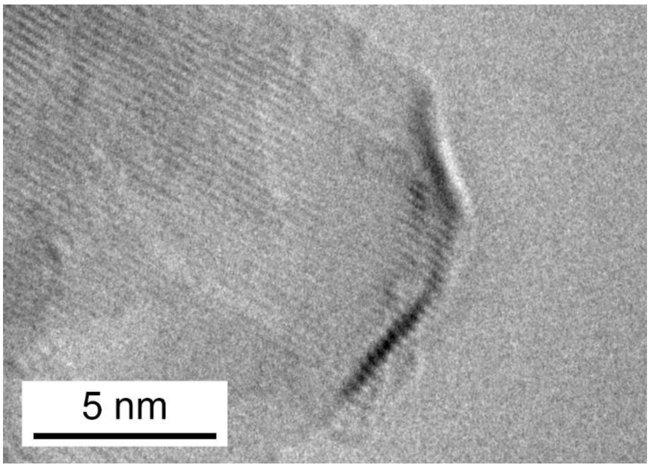

Figure S3. TEM image of the edge of one natural MoS_2 sheet.

4. Wide view of the as-synthesized MoS_2 through TEM.

Supplementary Fig. S4 is a TEM image of the as-synthesized MoS_2 with low magnification and wide view. Agglomerations of the as-synthesized MoS_2 can be observed clearly. It is believed that the agglomeration was caused by the solvent evaporation. It can be found that no particle-like morphology can be observed in the image. Therefore, it is proved that the as-synthesized MoS_2 is in the form of nanosheets instead of nanoparticles. Additionally, due to the ultrathin shape, individual nanosheets were very hard to be found.

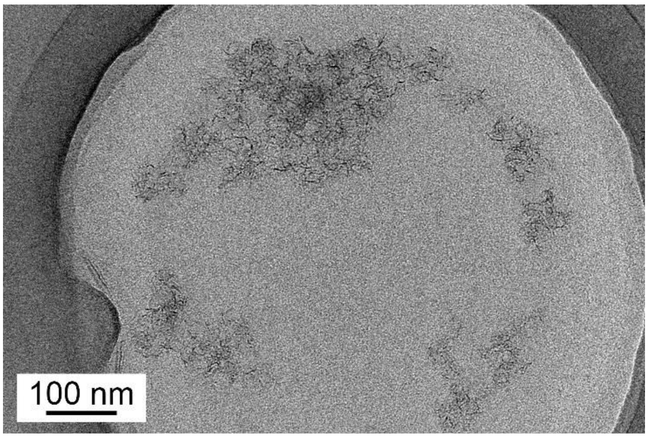

Figure S4. TEM image of agglomerations of the as-synthesized MoS_2 nanosheets.

5. The detection of oleylamine in the as-synthesized sample.

After the as-synthesized sample was washed with ethanol for 3 times and dried in vacuum, it was analysed with XPS and FTIR. In the C1s XPS spectrum, which is shown in Supplementary Fig. S5a, besides the peak at 284.8 eV, another peak at 285.2 eV can be identified. And the latter peak accords with R-CH_2-NH_2.[S3] In the infrared spectrum of the as-synthesized sample, which is displayed in Supplementary Fig. S5b, the broad NH_2 scissoring band at 1615 cm^{-1},[S4] which also appears in the spectrum of oleylamine, is very distinct. Considering that oleylamine is the only substance containing amine, it is indicated that oleylamine survives from the washing and still exists in the as-synthesized sample.

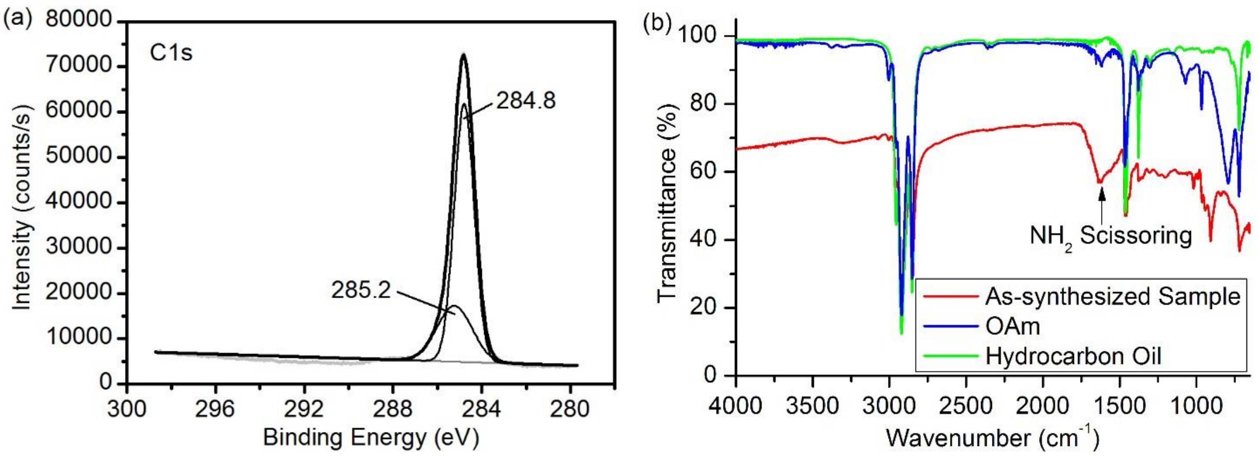

Figure S5. The detection of oleylamine (OAm) in the as-synthesized sample. (a) C1s XPS spectrum of the sample. (b) Infrared spectra of the as-synthesized sample, OAm and hydrocarbon oil.

6. Zeta potential of natural MoS_2 powders suspended in water.

To confirm the surface charge of MoS_2, natural MoS_2 powders were suspended in water with ultrasonic treatment. Then the zeta potential of the suspension was measured and is depicted in Fig. S6. The obtained zeta potential was 32.8 ±0.2 mV, proving that the surface of MoS_2 is negatively charged.

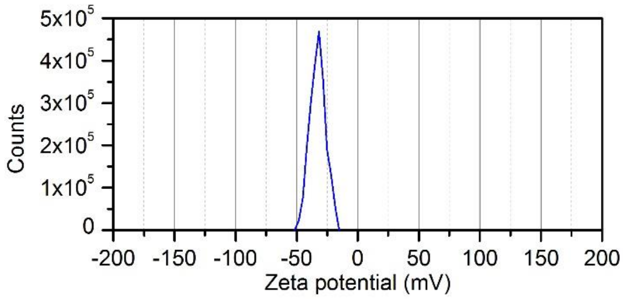

Figure S6. Zeta potential of natural MoS_2 powders suspended in water.

7. The characterizations of MoDDP before and after lubrication tests.

MoDDP is a commonly used Mo-contained organic lubricant additive and its molecular formula is presented in Supplementary Fig. S7a. Raman spectroscopy was used to analyse fresh or unused MoDDP and the spectrum is shown in Supplementary Fig. S7b, the C-H stretching bands near 2900 cm^{-1} are very obvious. Before the lubrication test with MoDDP failed, the test was stopped and the wear scar was analysed with Raman spectroscopy and EDS after ultrasonic cleaning with petroleum ether. The Raman spectrum is shown in Supplementary Fig. S7b. The peaks of E^1_{2g} and A_{1g} near 400 cm^{-1}, which do not exist in the spectrum of MoDDP, prove the creation of MoS_2. EDS result, which is provided in Supplementary Fig. S7c, also indicates that the elements of Mo and S exist in the wear scar. In addition, the lubricant after the tribological tests was collected, diluted with cyclohexane and dropped on a copper grid for TEM observation. Multilayer structured MoS_2 can be clearly observed (see Supplementary Fig. S7d). Above all, multilayer structured MoS_2 will be created when MoDDP is used as lubricant or lubricant additives. More detailed information about MoDDP can be found in Reference S5 & S6.

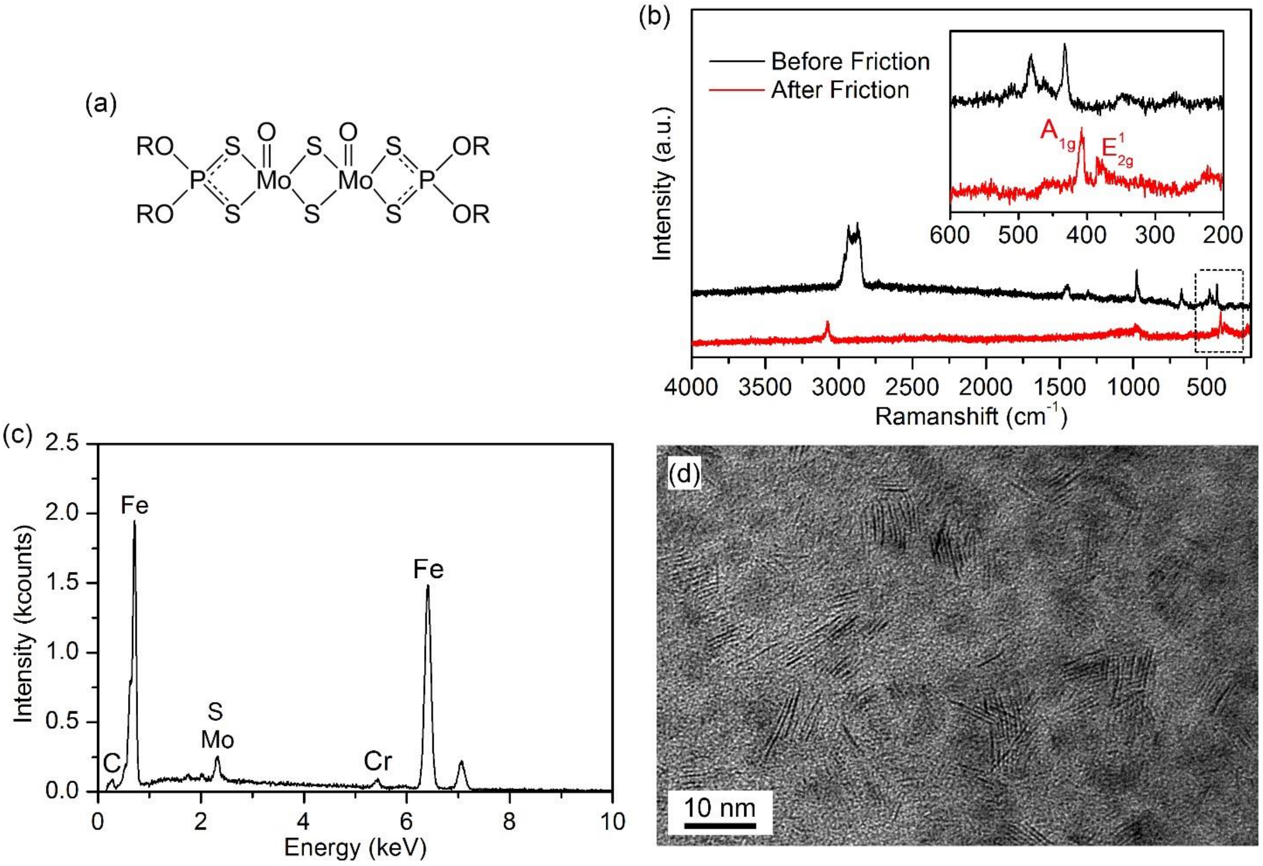

Figure S7. Characterizations of MoDDP before and after lubrication tests. (a) The molecular formula of MoDDP. (b) The Raman spectra of fresh MoDDP and the wear scar surface before the lubrication with MoDDP fails. (c) The EDS result of the wear scar surface before the lubrication fails. (d) The TEM image of the lubricant containing MoDDP after the tribological test.

8. The load-climbing tribological tests with various Mo concentration under different temperatures.

Tribological tests with various Mo weight fraction at different temperatures were performed. Tested additives were the as-synthesized MoS_2 nanosheets and MoDDP. The weight fraction of the element of Mo in each lubricant was ranged from 2 wt‰ to 6 wt‰ in the step of 2 wt‰. The test temperatures were 50 ℃ and 120 ℃ and the results are provided in Supplementary Fig. S8a and Supplementary Fig. S8b, respectively. Generally, it can be seen that the MoS_2 nanosheets perform much better than MoDDP. Under all the listed test conditions, the highest load with no seizure of the lubricant containing the as-

synthesized MoS_2 nanosheets is higher than 1000 N, while that of the MoDDP-contained lubricant is lower than 1000 N. When the temperature was 50 ℃ (see Supplementary Fig. S8a), the effective concentration of the Mo in the lubricant containing MoS_2 nanosheets is as low as 2 wt‰, with which the highest load with no seizure of the lubricant can exceed 2000 N. When the temperature was 120 ℃ (see Supplementary Fig. S8b), the effective concentration of the Mo in lubricant containing MoS_2 nanosheets is 4 wt‰.

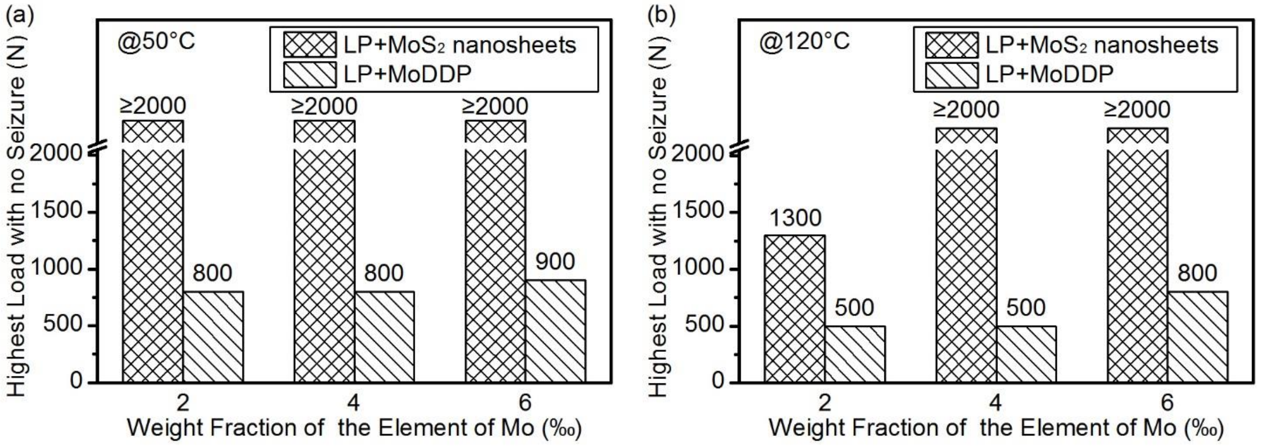

Figure S8. Highest load with no seizure of lubricants containing either MoS_2 nanosheets or MoDDP with different concentration of molybdenum (a) at 50 ℃ and (b) 120 ℃.

9. The introduction of TPPT and ZDDP.

TPPT and ZDDP are commonly used organic lubricant additives and their molecular formulas are displayed in Supplementary Fig. S9. When they work as lubricants or lubricant additives, both of them will form a tribofilm on the sliding surfaces to protect them. Detailed information about them can be found in Reference S7-S10.

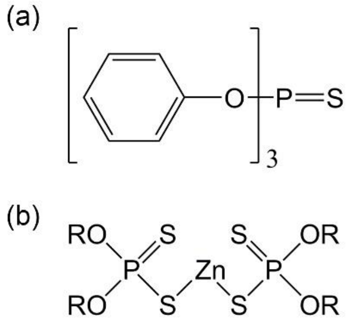

Figure S9. Molecular formulas of (a) TPPT and (b) ZDDP.

10. The load-climbing tribological tests of graphene, TPPT and ZDDP.

Load-climbing tests of lubricant containing graphene, TPPT and ZDDP were performed at the temperature of 120 ℃. Each of these lubricants is consisted of liquid paraffin and 1 wt% additive. The COF as a function of time of these tests are provided in Supplementary Fig. S10a. and the highest load with no seizure of these lubricants are shown in Supplementary Fig. S10b. The COF of the test of graphene rises abruptly when the load is 400 N, indicating that the highest load the lubricant can bear is 300 N. Coincidently, The lubricant containing TPPT has almost the same tribological performance with that of graphene. The lubrication with ZDDP fails when the load is 500 N, meaning that its limit is 400 N.

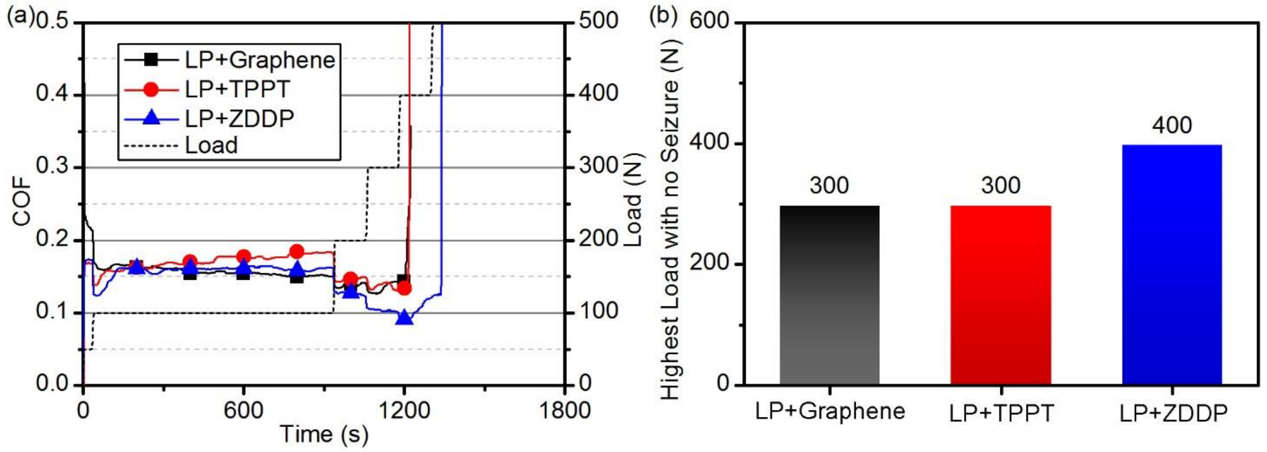

Figure S10. Extreme pressure tests of graphene, TPPT and ZDDP at 120 ℃. (a) COF as a function

of time of the tests of lubricants containing 1 wt% graphene, TPPT and ZDDP respectively. (b) Highest load with no seizure of these lubricants.

11. The calculation of pressure the lubricant containing MoDDP can bear.

As is found that, when the temperature is 120 ℃, the test of the lubricant containing MoDDP (Mo 6 wt‰) will stop when the load reaches 700 N. To obtain the highest pressure the lubricant can bear, the test was designedly stopped at 600 N, and the width of the wear scar (see Supplementary Fig. S11) was 0.89 ±0.04 mm. The pressure was calculated following the formula below and is about 0.96 GPa, which is smaller than that of the lubricant containing as-synthesized MoS_2 nanosheets under the same test conditions.

$$Pressure = \frac{Load}{\pi(\frac{d}{2})^2}$$

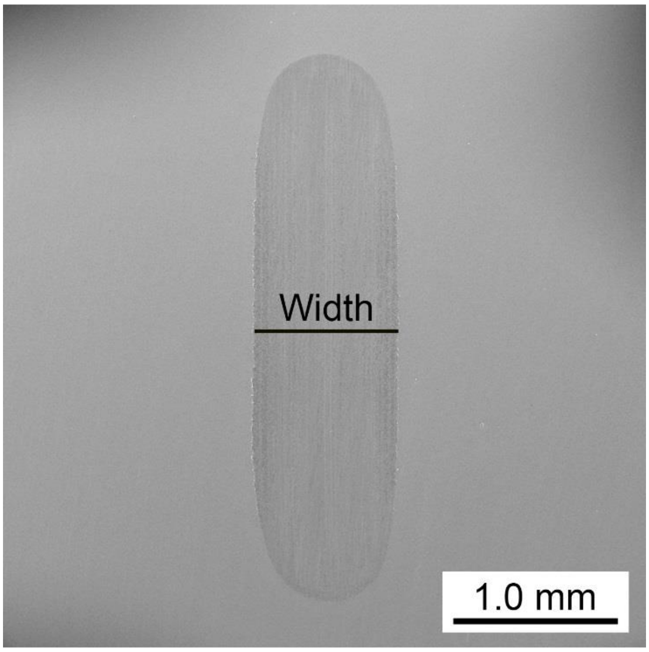

Figure S11. SEM image of the wear scar of test of the lubricant containing MoDDP (Mo 6 wt‰) stopped at 600 N.

References

S1. Wilson, J. A. & Yoffe, A. D. Transition metal dichalcogenides discussion and interpretation of observed optical, electrical and structural properties. *Adv. Phys.* **18**, 193-335 (1969).

S2. Eda, G. et al. Photoluminescence from Chemically Exfoliated MoS_2. *Nano Lett.* **11**, 5111-5116 (2011).

S3. Moulder, J. F., Stickle, W. F., Sobol, P. E., Bomben, K. D. *Handbook of X-Ray Photoelectron Spectroscopy* (Perkin-Elmer, Boca Raton, 1992).

S4. Stuart, B. *Infrared spectroscopy* (John Wiley & Sons, Inc., 2005).

S5. Yamamoto, Y., Gondo, S., Kamakura, T. & Tanaka, N. Frictional characteristics of molybdenum dithiophosphates. *Wear* **112**, 79-87 (1986).

S6. Yamamoto, Y. & Gondo, S. Friction and wear characteristics of molybdenum dithiocarbamate and molybdenum dithiophosphate. *Tribol. Trans.* **32**, 251-257 (1989).

S7. Mangolini, F., Rossi, A. & Spencer, N. D. Reactivity of triphenyl phosphorothionate in lubricant oil solution. *Tribol. Lett.* **35**, 31-43 (2009).

S8. Mangolini, F., Rossi, A. & Spencer, N. D. Chemical reactivity of triphenyl phosphorothionate (TPPT) with iron: An ATR/FT-IR and XPS investigation. *J. Phys. Chem. C.* **115**, 1339-1354 (2011).

S9. Martin, J. M. Antiwear mechanisms of zinc dithiophosphate: A chemical hardness approach. *Tribol. Lett.* **6**, 1-8 (1999).

S10. Spikes, H. The history and mechanisms of ZDDP. *Tribol. Lett.* **17**, 469-489 (2004).
